# BET inhibition is an effective approach against KRAS-driven PDAC and NSCLC

**DOI:** 10.18632/oncotarget.24648

**Published:** 2018-04-10

**Authors:** Toni Jauset, Daniel Massó-Vallés, Sandra Martínez-Martín, Marie-Eve Beaulieu, Laia Foradada, Francesco Paolo Fiorentino, Jun Yokota, Bernard Haendler, Stephan Siegel, Jonathan R. Whitfield, Laura Soucek

**Affiliations:** ^1^ Vall d’Hebron Institute of Oncology (VHIO), Edifici Cellex, Hospital Vall d’Hebron, Barcelona, Spain; ^2^ Department of Biochemistry and Molecular Biology, Universitat Autònoma de Barcelona, Bellaterra, Barcelona, Spain; ^3^ Peptomyc S.L., Edifici Cellex, Hospital Vall d’Hebron, Barcelona, Spain; ^4^ Kitos Biotech srls, Porto Conte Ricerche, Alghero, Italy; ^5^ Department of Biomedical Sciences, University of Sassari, Sassari, Italy; ^6^ Genomics and Epigenomics of Cancer Prediction Program, Institut d’Investigació Germans Trias I Pujol (IGTP), Campus Can Ruti, Barcelona, Spain; ^7^ Drug Discovery, Bayer AG, Berlin, Germany; ^8^ Institució Catalana de Recerca i Estudis Avançats (ICREA), Barcelona, Spain

**Keywords:** BET inhibition, MYC, PDAC, NSCLC

## Abstract

Effectively treating KRAS-driven tumors remains an unsolved challenge. The inhibition of downstream signaling effectors is a way of overcoming the issue of direct targeting of mutant KRAS, which has shown limited efficacy so far. Bromodomain and Extra-Terminal (BET) protein inhibition has displayed anti-tumor activity in a wide range of cancers, including KRAS-driven malignancies. Here, we preclinically evaluate the effect of BET inhibition making use of a new BET inhibitor, BAY 1238097, against Pancreatic Ductal Adenocarcinoma (PDAC) and Non-Small Cell Lung Cancer (NSCLC) models harboring RAS mutations both *in vivo* and *in vitro*. Our results demonstrate that BET inhibition displays significant therapeutic impact in genetic mouse models of KRAS-driven PDAC and NSCLC, reducing both tumor area and tumor grade. The same approach also causes a significant reduction in cell number of a panel of RAS-mutated human cancer cell lines (8 PDAC and 6 NSCLC). In this context, we demonstrate that while BET inhibition by BAY 1238097 decreases MYC expression in some cell lines, at least in PDAC cells its anti-tumorigenic effect is independent of MYC regulation. Together, these studies reinforce the use of BET inhibition and prompt the optimization of more efficient and less toxic BET inhibitors for the treatment of KRAS-driven malignancies, which are in urgent therapeutic need.

## INTRODUCTION

Mutation in the *KRAS* oncogene is one of the most frequent events in human cancers. In Non-Small Cell Lung Cancer (NSCLC), the main lung cancer subtype that accounts for the highest number of cancer-related deaths, *KRAS* is mutated in 30% of the cases [1]. In Pancreatic Ductal Adenocarcinoma (PDAC), which presents the lowest survival rates among all cancers, 90% of the tumors harbor *KRAS* mutations [2]. Because of its high prevalence and relevance, KRAS has been extensively studied with the ultimate objective of finding a safe and effective therapeutic strategy for patients presenting KRAS-driven tumors. Despite the knowledge acquired over the past years and the new therapeutic technologies, treating KRAS-mutated cancers still remains a major health issue.

Indeed, targeting KRAS has proven challenging at multiple levels and different strategies have been explored: inhibiting *KRAS* translation by interfering with its messenger RNA, impairing KRAS processing using farnesyltransferase inhibitors, directly targeting the KRAS protein by peptide inhibitors or instructing the immune system against mutant KRAS [3]. However, no clinical trial based on these approaches has so far demonstrated convincing anti-tumorigenic activity [4, 5]. Others have adopted a different approach by targeting downstream RAS effectors such as mTOR, PI3K, Akt or MEK. Although trials are ongoing, these drugs have not proven to be effective against RAS-driven cancers in patients thus far [6]. In this context, some groups have recently demonstrated the therapeutic potential of Bromodomain and Extra-terminal (BET) protein inhibition in NSCLC and PDAC preclinical models [7–12]. Bromodomains recognize the N-terminal acetylated lysines on histones and recruit chromatin-regulating factors on promoters and enhancers to control gene expression. The key role of BET bromodomains in cancer initiation and maintenance has been highlighted by the development of small molecule BET bromodomain inhibitors [13]. Such inhibitors prevent the interaction between bromodomains and acetylated lysines, displaying significant anti-tumorigenic activity by regulating key engines of tumorigenesis like MYC [14, 15]. While the efficacy of BET inhibitors expands to a wide range of cancers, evidences of *de novo* and acquired resistance to some compounds have already been observed [16–18]. Moreover, there is an increasing concern about the potential toxicity that BET inhibitors might display in normal tissues versus cancer cells [19].

BAY 1238097 is a new BET bromodomain inhibitor with potent anti-tumor activity in B cell lymphoma and melanoma models, both *in vitro* and *in vivo* [20, 21], which has recently been evaluated in a phase I dose-escalation trial in patients with advanced malignancies [22]. In the present study, we have determined the anti-tumorigenic impact of BET inhibition against two different immunocompetent KRAS-driven mouse models. We have then expanded the study to human PDAC and NSCLC cell lines, to investigate whether the anti-tumorigenic activity of the compound is dependent on MYC downregulation in a human setting. Our results indicate that BET bromodomain inhibition might be an effective therapeutic option for patients harboring KRAS-mutated PDAC and NSCLC, even independently of MYC regulation, as seems to be the case in PDAC.

## RESULTS

### BET inhibition is effective against KRAS-driven NSCLC and PDAC mouse models

In order to preclinically assess the *in vivo* efficacy of BET inhibition by BAY 1238097 in an immunocompetent context, we made use of two well-characterized KRAS-driven genetically engineered mouse models of PDAC (*LSL-Kras*^*G12D*^*;Pdx1-Cre;p53*^*ER/ER*^) and NSCLC (*LSL-Kras*^*G12D*^*;p53*^*ER/ER*^) [23, 24]. In both models, the constitutively-active *Kras*^*G12D*^ mutant allele is expressed from the endogenous *Kras* locus after CRE-mediated recombination. Briefly, constitutive *Kras*^*G12D*^ transcription is prevented by a *loxP-STOP-loxP* (*LSL*) cassette. Expression of CRE recombinase excises the *LSL* cassette, activating the expression of *Kras*^*G12D*^ and consequently triggering tumorigenesis in a tissue-specific manner. In the PDAC model, *Cre* recombinase is placed under the control of the *Pdx1* promoter, which is activated in progenitor cells of mouse pancreas [23], while in the NSCLC model, adenocarcinomas are generated focally in the lung epithelium by delivering CRE recombinase through intranasal instillation of adenoviruses (Ad-*Cre*) [24]. Additionally, in both mouse models, the *p53* wild type alleles were substituted by an inactive form of P53 (*p53*^*ER/ER*^), which accelerates tumor progression to better recapitulate the aggressiveness and heterogeneity of human tumors [25, 26].

**Figure 1:**
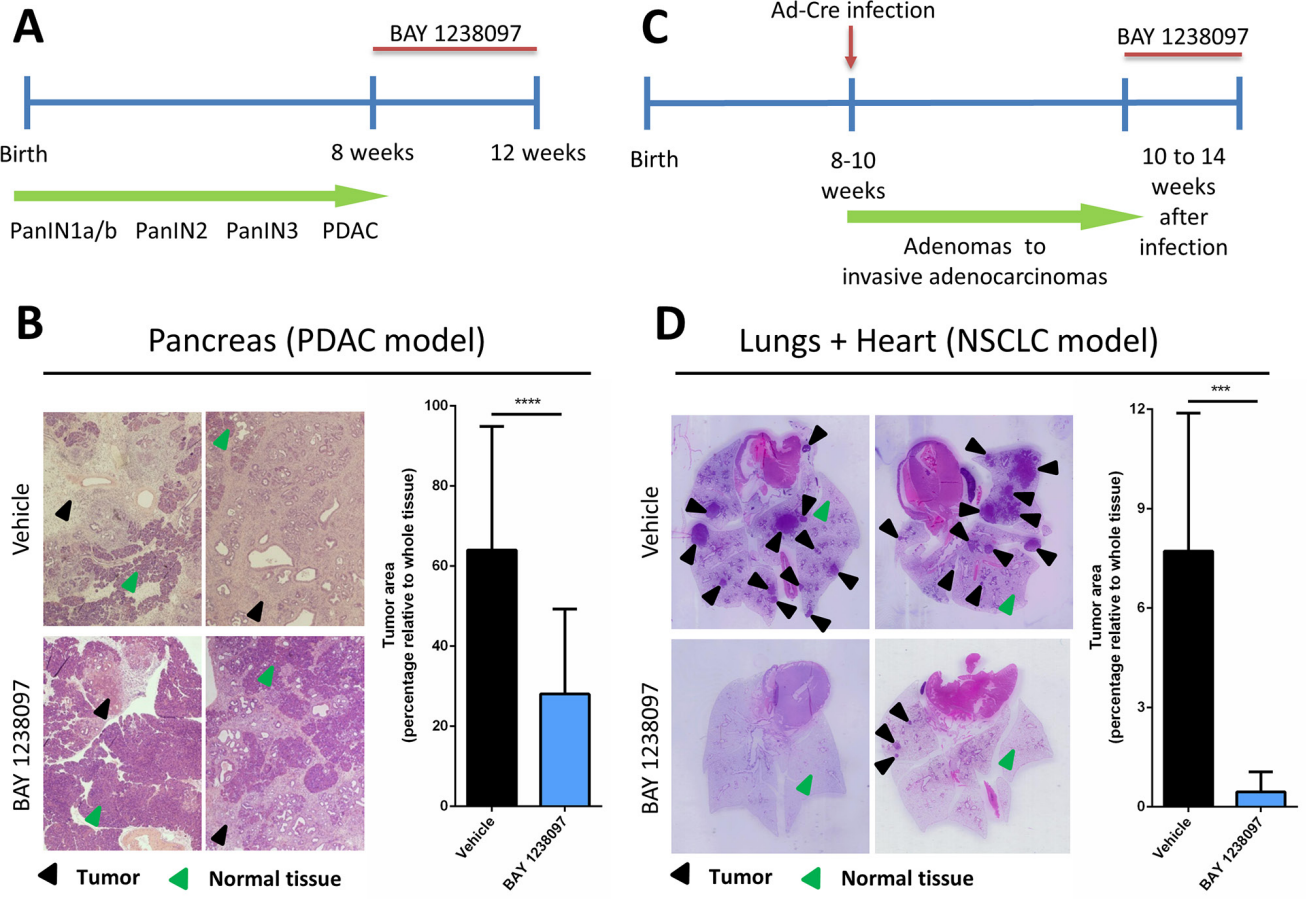
Treatment with BAY 1238097 reduces tumor burden in genetic mouse models of KRAS-driven PDAC and NSCLC Timeline of the therapeutic intervention with BAY 1238097 in **(A)** PDAC and **(C)** NSCLC are represented. In the pancreas model, tumors were allowed to evolve for 8 weeks to reach the PDAC stage, while in the lung model tumors developed for 10 weeks to adenocarcinomas. Treatment was then administered for 4 weeks. Therapeutic impact of BAY 1238097 after 4 weeks of treatment in **(B)** PDAC and **(D)** NSCLC is shown. Representative images of H&E-stained sections from lungs and pancreas of each model in vehicle (upper panels) and BAY 1238097 treated samples (lower panels) are shown. Black arrows indicate tumorigenic tissue and green arrows indicate normal tissue. Graphs show quantification of tumor burden as the percentage of tumor area relative to the whole tissue (tumor+normal tissue). Means and standard deviations are represented. For statistical analysis of the data, two-tailed unpaired *t*-tests between groups were performed; p<0.0001 (^****^) and p=0.0002 (^***^).

To investigate the therapeutic impact of BET inhibition by BAY 1238097 in PDAC, we treated tumor-bearing 8 week-old *LSL-Kras*^*G12D*^*;Pdx1-Cre;p53*^*ER/ER*^ mice. The animals were treated for 4 weeks with 35 mg/kg of BAY 1238097 (maximum tolerated dose in mice) or with vehicle via oral gavage (Figure 1A), pancreata were collected and tumor burden evaluated by hematoxylin and eosin (H&E) staining (Supplementary Figure 1A). Histology analysis revealed a dramatic reduction of the tumor area relative to whole tissue in the treated samples compared to the control counterparts (28±21% vs 64±31%) (Figure 1B). In addition, while the tumors of the control group were mainly graded as PDACs, in the treated cohort tumors of lower grades (PanIN2 and PanIN3) were prevalent over PDAC regions (Table 1A). During the course of the treatment, weight was recorded twice a week as a general read-out of animal health. All mice showed a progressive increase of weight for both treated and control groups, although control mice gained more weight compared to the treated ones (10.12±4.04% vs 5.45±4.39%, at the endpoint) (Supplementary Figure 2A), indicative of mild drug toxicity.

**Table 1:** Mice treated with BAY 1238097 presented lower tumor grades compared to untreated animals

A		
PDAC model		
Sample ID	Group	Grades Observed
PC-1	Vehicle	PDAC
PC-2	Vehicle	PDAC
PC-3	Vehicle	PDAC
PC-4	Vehicle	PDAC
PC-5	Vehicle	PDAC
PC-6	Vehicle	PanIN3, PDAC
PC-7	Vehicle	PDAC
PC-8	Vehicle	PDAC
PC-9	Vehicle	PDAC
PC-10	Vehicle	PDAC
PC-11	Vehicle	PDAC
PT-1	Treated	PanIN2, PanIN3, PDAC
PT-2	Treated	PanIN2, PanIN3, PDAC
PT-3	Treated	PanIN2, PanIN3, PDAC
PT-4	Treated	PanIN2, PanIN3, PDAC
PT-5	Treated	PanIN3, PDAC
PT-6	Treated	PanIN2, PanIN3, PDAC
PT-7	Treated	PanIN2, PanIN3, PDAC
PT-8	Treated	PanIN3, PDAC
PT-9	Treated	PanIN2, PanIN3, PDAC
PT-10	Treated	PanIN2, PanIN3, PDAC

B		
NSCLC model		
Sample ID	Group	Grades Observed
LC-1	Vehicle	Adenocarcinoma
LC-2	Vehicle	Adenocarcinoma
LC-3	Vehicle	Adenocarcinoma
LC-4	Vehicle	Adenocarcinoma
LC-5	Vehicle	Adenocarcinoma
LC-6	Vehicle	Adenocarcinoma
LC-7	Vehicle	Adenocarcinoma
LC-8	Vehicle	Adenocarcinoma
LT-1	Treated	-
LT-2	Treated	AAH
LT-3	Treated	-
LT-4	Treated	AAH
LT-5	Treated	AAH
LT-6	Treated	-
LT-7	Treated	AAH
LT-8	Treated	AAH

(A) Presence of pancreatic intraepithelial lesions (PanIN) of grade 2 and 3 and PDACs were evaluated in the pancreata sections of the PDAC model (Supplementary Figure 1A). (B) In the NSCLC model (Supplementary Figure 1B), presence of atypical adenomatous hyperplasias (AAH), large adenomas and adenocarcinomas were evaluated. “-“ represents tumor-free sections.

In parallel, to assess the efficacy of BAY 1238097 in NSCLC, lung tumors were induced in 8- to 10-week old *LSL-Kras*^*G12D*^*;p53*^*ER/ER*^ mice by Ad-*Cre* administration. Tumors were allowed to develop for 10 weeks and then animals were treated with BAY 1238097 or vehicle for 4 weeks (Figure 1C). At treatment endpoint, lung tissues were harvested and H&E-stained (Supplementary Figure 1B). Quantification of tumor area showed an even more striking effect than in PDAC: both tumor burden relative to whole lung epithelium and tumor number were dramatically reduced by BET inhibitor treatment (7.72±4.17% vs 0.45±0.60% and an average of 10±3 vs 2±1 tumors per animal, in untreated versus treated animals respectively) (Figure 1D). Strikingly, the lungs of 3 out of 8 treated animals were completely tumor-free and the remaining 5 presented atypical adenomatous hyperplasia, while all the untreated mice showed presence of multiple adenocarcinomas (Table 1B). However, while the weight of control mice remained stable during the treatment window, the treated counterparts displayed a clear decrease (gain of 2.90±3.05 vs loss of 5.97±3.45 at the endpoint) (Supplementary Figure 2B). In this case and in accordance with the protocol approved by our ethical committee, 3 out of 8 treated animals that experienced more than 10% weight-loss skipped the treatment until weight recovery. Notably, all mice recovered rapidly - in only one or two days - indicating that the mild toxicity of the compound is quickly reversible. However, in this context, it is worth noting that 2 of the 5 treated animals still harboring tumors at the end of the experiments had been subject to a “drug holiday” due to weight loss, indicating that continuous treatment with the compound might be potentially more effective. Nevertheless, mice given suboptimal (interrupted) treatment still presented a clear reduction in tumor burden when compared to untreated animals (0.77±0.91% vs 7.72±4.17%).

Although these results suggest that NSCLC responds more effectively than PDAC to the treatment with the BET inhibitor, this therapeutic strategy seems to be a good therapeutic option against both KRAS-mutated diseases.

### BET inhibition downregulates MYC in the KRAS-driven PDAC and NSCLC mouse cells

BET bromodomain inhibition has been reported to be a potential strategy to inhibit MYC [14], a central node of tumorigenesis. The BET inhibitor JQ1 has shown an anti-tumorigenic effect by suppressing MYC expression in both PDAC [12] and NSCLC [9]. In contrast, others have shown that BET inhibition is able to abolish tumorigenesis independently of MYC regulation [7, 8].

**Figure 2:**
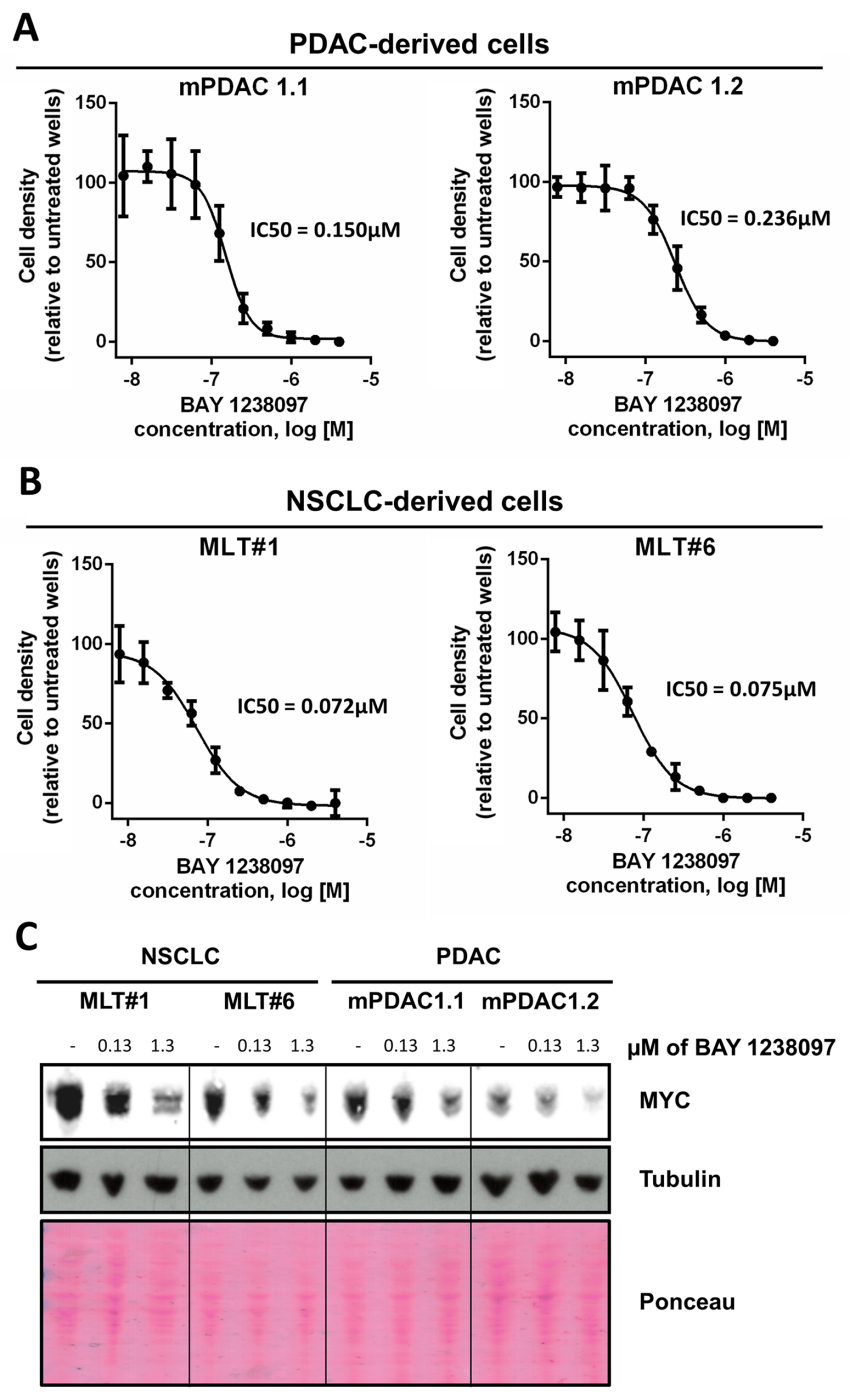
PDAC- and NSCLC-derived cell lines from the *LSL-Kras*^*G12D*^*;Pdx1-Cre;p53*^*ER/ER*^ and *LSL-KrasG12D;p53ER/ER* genetic mouse models showed IC50s within the nanomolar range and a dose-dependent MYC decrease in response to BAY 1238097 2 cell lines derived from each model were treated with varying concentrations of BAY 1238097 for 3 days. Cell were then fixed, stained with crystal violet and absorbance was quantified. IC50s were determined for cells derived from the PDAC **(A)** and NSCLC **(B)** models. Means and standard deviations are indicated. **(C)** A representative Western Blot is shown of all 4 cell lines treated with 0.13 and 1.3 μM of BAY 1238097 for 24 hours. Tubulin and Ponceau S are provided as loading controls.

To determine the potential regulation of MYC in murine PDAC and NSCLC tumors treated with BAY 1238097, we generated two cell lines from each tumor type (PDAC: mPDAC 1.1 and mPDAC 1.2; NSCLC: MLT#1 and MLT#6) (Supplementary Figure 3) and treated them *in vitro* for 3 days with the compound. In these conditions, BAY 1238097 caused a reduction in cell number at the nanomolar range in both PDAC and NSCLC cell lines (Figure 2A–2B). Interestingly, similarly to their *in vivo* counterparts, the 2 NSCLC-derived cell lines showed a higher sensitivity to BAY 1238097 compared to the PDAC-derived cell lines (IC50 of 0.072 and 0.075 μM vs 0.236 and 0.150 μM respectively).

To check for MYC levels, protein extracts were obtained after 24 hours of treatment at 2 different concentrations of the compound (one corresponding to the average of all 4 IC50s, and the other one 10-fold higher). Western Blot analysis showed a dose-dependent decrease of MYC protein levels in all 4 cell lines (Figure 2C), implying that BAY 1238097 is able to regulate MYC expression in these experimental models.

### MYC downregulation correlates with increased sensitivity to BET inhibition in NSCLC but not in PDAC human cell lines

**Figure 3:**
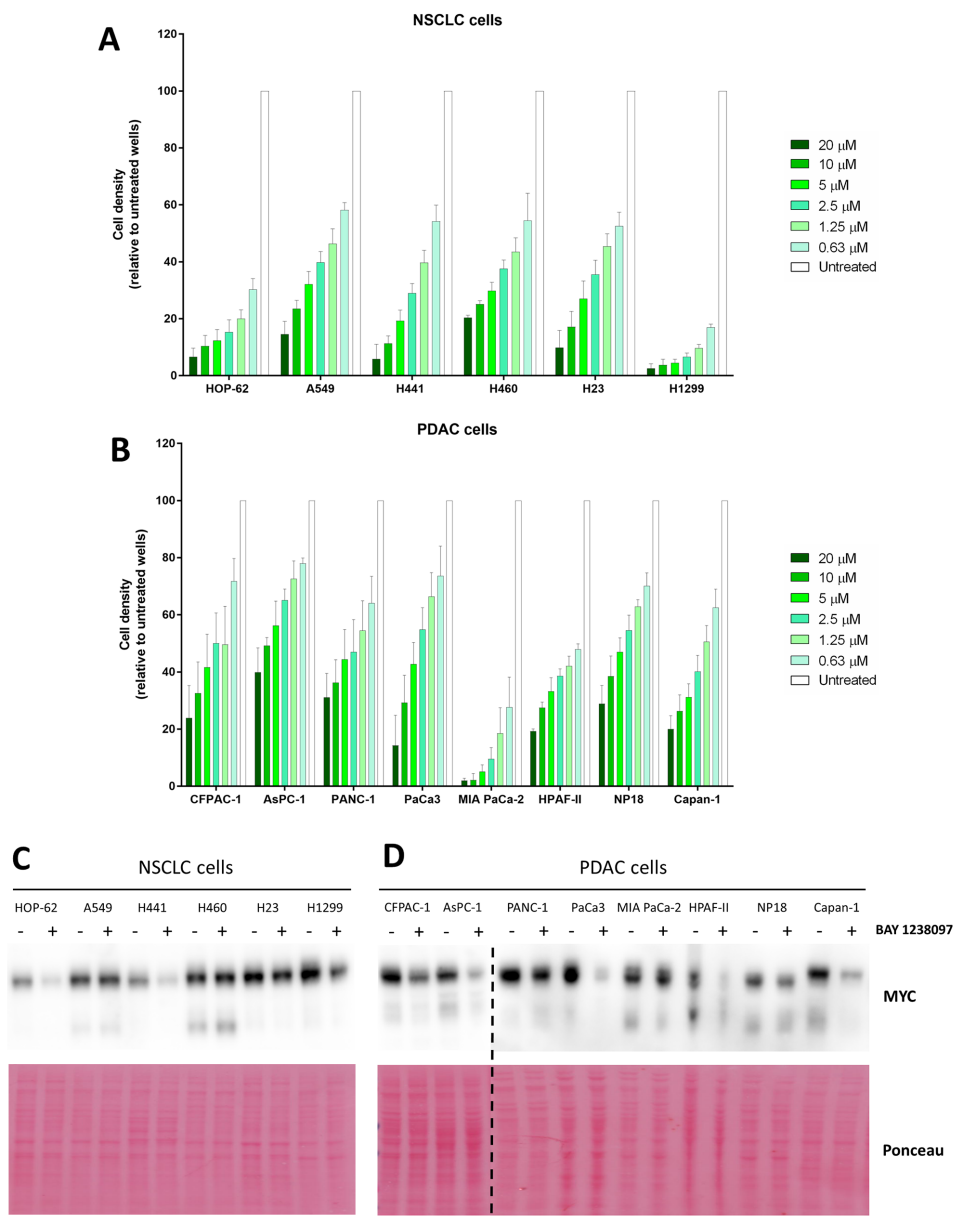
Human PDAC and NSCLC cell lines respond to BAY 1238097 and show variable MYC downregulation upon BET inhibition Cell density (relative to untreated controls) of **(A)** NSCLC and **(B)** PDAC cell lines after 3 days of treatment at 0.63, 1.25, 2.5, 5, 10 and 20 μM was calculated using crystal violet staining and quantification of absorbance. A two-tailed unpaired *t*-test was performed for statistical analysis of each concentration vs. the corresponding untreated control; all comparisons show statistically significant differences (p<0.0001). Western Blots of **(C)** NSCLC and **(D)** PDAC cell lines untreated (-) or treated (+) with 0.63 μM of BAY 1238097 were probed to detect MYC (n=2). Ponceau S staining was used as protein loading control. The dotted line indicates separate blots. Quantification of MYC downregulation is represented in Supplementary Figure 4.

To determine whether the therapeutic effect of BAY 1238097 extends also to human tumors, a panel of NSCLC (H23, A549, H1299, H460, HOP-62 and H441) and PDAC (NP18, PANC-1, PaCa3, MIA PaCa-2, HPAF-II, AsPC-1, Capan-1 and CFPAC-1) human cell lines were treated with the compound. Of note, all these cell lines harbor different activating mutations in the *KRAS* oncogene, with the exception of H1299 that is *NRAS* mutated, and most of them mutations in p53 as well (Supplementary Table 1). Importantly, simultaneous *LKB1* and *KRAS* mutations in lung adenocarcinoma cell lines were previously shown to prevent MYC downregulation and sensitivity in response to BET inhibition [9]. Thus, we included 3 *LKB1*-mutated cell lines in our NSCLC panel (A549, H23 and H460) (Supplementary Table 1 A). With this comprehensive experimental system, we investigated the sensitivity of each cell line to the BET inhibitor, treating cells with different concentrations of the compound (0.63, 1.25, 2.5, 5, 10, 20 μM) for 3 days. Even though all human cell lines responded to 10 μM of BAY 1238097 displaying a reduction of at least 50% of cell density, various degrees of sensitivity were observed (Figure 3A–3B). Overall, NSCLC cell lines presented higher sensitivity than PDAC cell lines, as previously observed in the mouse models both *in vivo* and *in vitro*. Among PDAC and NSCLC cell lines, MIA PaCa-2 and H1299 showed the highest response respectively.

In order to establish whether there was any correlation between efficacy of the BET inhibitor and regulation of MYC protein levels, all cell lines were treated with 0.63 μM of the compound (the lowest concentration tested, which was effective against the most sensitive cell lines) and protein lysates were collected 24 hours later for Western Blot analysis. Half of the analyzed cell lines (7 out of the 14: CFPAC-1, AsPC-1, PaCa3, HPAF-II, Capan-1, HOP-62, and H441) displayed a reduction of MYC to less than 50% compared to the untreated cell lines (Figure 3C–3D and Supplementary Figure 4).

Consistently with previously published data [9], while the cell lines harboring wild type LKB1 in the NSCLC panel (HOP-62, H1299 and H441) showed a clear reduction in MYC levels, the cell lines with the mutated tumor suppressor (A549, H460 and H23) did not show a comparable regulation.

Six cell lines of our PDAC panel (CFPAC-1, AsPC-1, PANC-1, MIA PaCa-2, HPAF-II and Capan-1) are present in the Catalogue of Somatic Mutations in Cancer (COSMIC) and appear to harbor wild type LKB1 alleles (Supplementary Table 1 B). In those cells, we observed reduction of MYC protein levels, with the notable exception of MIA PaCa-2 that, despite harboring wild type LKB1, did not show any significant MYC downregulation (Figure 3D and Supplementary Figure 4). The other 2 PDAC cell lines, whose LKB1 status is not present in COSMIC, responded to the inhibitor with a moderate (NP18) or strong (PaCa3) reduction in MYC levels too.

**Figure 4:**
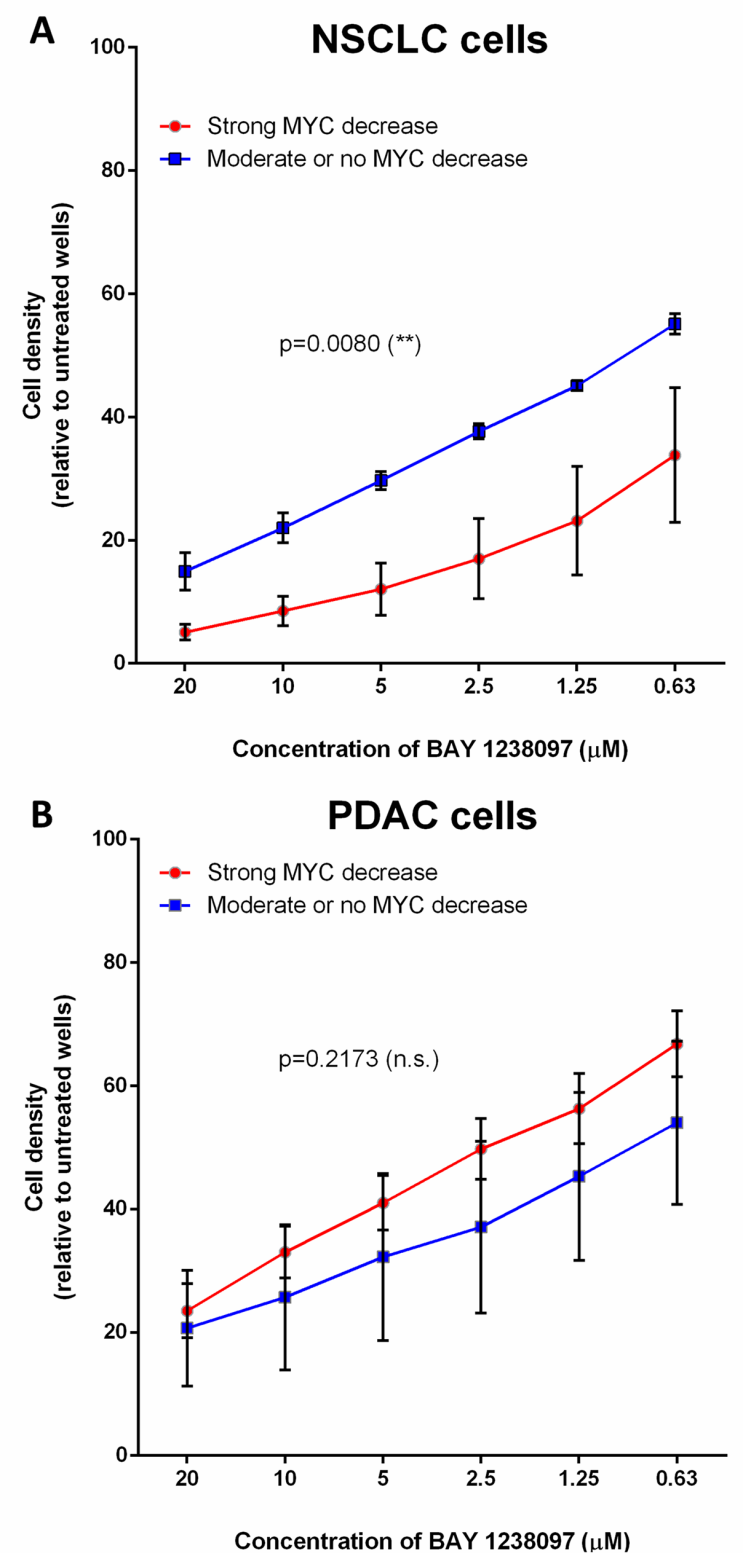
The higher response of NSCLC cell lines to BET inhibition correlates with MYC downregulation, while PDAC cells display MYC-independent sensitivity to the compound Cell lines were grouped according to MYC downregulation upon BAY 1238097 treatment (refer to Supplementary Figure 4). In **(A)**, A549, H460 and H23 display no change or a moderate MYC decrease (blue line), while HOP-62, H441 and H1299 show clear MYC downregulation (red line). In **(B)**, PANC1, MIA PaCa-2 and NP18 show minimum MYC changes (blue line), while CFPAC-1, AsPC-1, PaCa3, HPAF-II and Capan-1 display a clear reduction in MYC levels (red line). Means of the group of cell lines and standard errors of the mean (SEM) of these values are represented. For statistical analysis of the data, the area under the curve and SEM were calculated with GraphPad Prism 7 and two-tailed unpaired *t*-test between groups was performed; p = 0.0080 (^**^) and p=0.2173 (non-significant; n.s.).

Interestingly, within the NSCLC cell lines, LKB1-wild type cells that show a clear decrease of MYC protein upon BAY 1238097 treatment also display a significantly higher sensitivity to the inhibitor than the cell lines in which MYC levels remain unchanged or slightly decreased (Figure 4A). However, a similar analysis shows that this correlation is not true in PDAC cells, where the sensitivity to the inhibitor does not mirror MYC downregulation (Figure 4B).

In summary, in our study, BET inhibition by BAY 1238097 showed remarkable efficacy against NSCLC and PDAC *in vivo*, significantly reducing tumor burden in genetic mouse models of KRAS-driven tumors. The BET inhibitor also showed efficacy *in vitro* in RAS-mutated NSCLC and PDAC human cell lines. The degree of response partially correlated with a downregulation of MYC in NSCLC, but not in PDAC, implying the existence of other molecular effectors that will need to be further investigated. Taken together, these results demonstrate that BET bromodomain inhibition may be an effective anti-cancer approach against RAS-mutated PDAC and NSCLC, which are in urgent need of new therapeutic options.

## DISCUSSION

In cancer generally, and particularly in PDAC and NSCLC, *KRAS* is one of the most frequently mutated oncogenes. Despite the increasing number of studies and the technological advance in the generation of more effective therapies, direct KRAS inhibition has proven to be extremely challenging and, when tested in the clinic, provided an insufficient therapeutic index in patients [5, 27]. Hence, many groups have instead put their efforts into targeting Ras indirectly, through the inhibition of its multiple effector pathways. Among those, BET bromodomains might constitute a well characterized and tractable target. BAY 1238097 is a new BET inhibitor that has been tested against lymphoma [20] and, more recently, melanoma [21], but its use against KRAS-mutated tumors has not been yet investigated. Here, we used genetic mouse models of KRAS-driven NSCLC and PDAC to underpin the efficacy of BET inhibitors against KRAS-driven malignancies and preclinically validate their use in tumors presenting mutations in KRAS. These models develop tissue-specific lesions that arise from a single alteration in KRAS and evolve, through acquisition of additional mutations, until the development of macroscopic adenocarcinomas, in a process that resembles the natural development of cancer in humans. Importantly, these experimental animals possess a fully operative immune system, a key element to evaluate the response to therapies, both in terms of efficacy and off-target toxicity. In addition, in order to enhance the tumor heterogeneity and genetic complexity, which constitute notable features of human cancers, we adopted models in which both endogenous copies of the p53 tumor suppressor have been substituted by an impaired p53 (p53^ER/ER^), allowing for accelerated mutational rate and the development of more aggressive and heterogeneous tumors [25, 26]. In both NSCLC and PDAC genetic mouse models, BAY 1238097 caused a remarkable reduction of the tumor burden, not only decreasing the tumor area, but also reducing the tumor grade. Interestingly, the therapeutic impact was more dramatic in the NSCLC model than in PDAC, an observation that has been consistent across the subsequent *in vitro* studies both in mice and human cells. This suggests that an intrinsic feature of NSCLC cells might further sensitize them compared to PDAC cells. In addition, *in vivo*, the lower sensitivity of PDAC could also be related to the stromal fibro-inflammatory reaction characteristic of these tumors, which may result in lower drug penetration and, therefore, reduced efficacy, as previously reported for other drugs [28, 29].

Despite these slight differences, the overall efficacy of BET inhibition in these two models indicates that this can represent an effective therapeutic approach against both KRAS-mutated cancers.

The effectivity of BET bromodomain inhibitors, especially JQ1, has been linked to the downregulation of MYC [14, 15]. However, in some cases, BET inhibition may exert its anti-tumorigenic effect independently of MYC inhibition [30]. To investigate this aspect in a RAS-mutated context, we made use of both mouse-derived and human cancer cell lines. BET inhibition does indeed downregulate MYC in most cells. However, MYC remained largely unchanged upon BAY 1238097 treatment in a subset of 3 NSCLC cell lines. Notably, these are the only 3 cell lines presenting mutations in LKB1, which has been previously described to confer resistance to BET-mediated MYC inhibition [9]. Hence, even if LKB1 mutation does not confer resistance to BAY 1238097, our results reinforce the correlation of wild type LKB1 and the susceptibility to MYC downregulation upon BET inhibition, which appears to predispose cells to a higher anti-tumorigenic effect compared to impaired MYC downregulation due to LKB1 mutations, at least in NSCLC. In PDAC, all the cell lines whose LKB1 status is known (6/8) harbor the wild type tumor suppressor and, consistently with the results in NSCLC, upon treatment with the BET inhibitor, downregulation of MYC is preponderant. However, the sensitivity of PDAC cell lines is lower than that of NSCLC cell lines, which implies that other molecular mechanisms besides MYC regulation might play a key role in mediating the anti-tumorigenic effect of BET inhibition. Indeed, in contrast to NSCLC, the degree of MYC regulation in our PDAC cell lines does not correlate with the response to the inhibitor. This phenomenon is best exemplified in MIA PaCa-2 and PaCa3: while MIAPaCa-2 cells barely display changes in MYC upon treatment, they show the strongest response to the compound; in contrast, PaCa3 cells, which display the most dramatic reduction in MYC levels upon treatment, are one of the least sensitive PDAC cell lines.

Therefore, our study suggests that the MYC-mediated sensitivity of KRAS-mutated cancer cells to BET inhibition is highly context dependent and that such effectivity relies on more than one molecular mechanism. More investigation will need to be undertaken to identify the key molecular players, other than MYC, that contribute to the increased sensitivity to BET inhibitors.

Finally, it should be noted that, unfortunately, a recent Phase I clinical trial study based on BAY 1238097 was discontinued due to dose-limiting toxicity in patients [22], indicating that more investigation is needed to explore and identify BET inhibitors with limited side effects that retain anti-tumor efficacy within an acceptable therapeutic window.

Other BET bromodomain inhibitors with different chemical scaffolds are currently being tested in the clinic for various haematological and solid tumor types, including pancreatic ductal adenocarcinoma and NSCLC. The most advanced ones include CPI-0610, GS-5829, GSK525762, INCB054329, INCB057643 and BMS-986158, which are currently being evaluated in Phase I/II or Phase II studies. It remains to be determined whether monotherapy with BET bromodomain inhibitors will show significant efficacy within an acceptable therapeutic window or whether combinations with other anti-cancer drugs will be required to increase the therapeutic impact of BET inhibitors [31, 32].

## MATERIALS AND METHODS

### BAY 1238097 preparation

Chemical structure and synthesis of BAY 1238097 have been described [21]. Lyophilized BAY 1238097 was stored at room temperature in a dry environment protected from light. Fresh working solutions (3.5g/L) were prepared weekly by stirring and resuspending the powder in 0.9% NaCl at 50°C for 1 hour. HCl was added dropwise until a clear solution was obtained and pH was finally adjusted at 3.6. The resulting solution was filtered through a 0.22μm filter and stored at room temperature protected from light for a maximum of 7 days for animal studies or frozen at -20°C for a maximum of 1 month for *in vitro* studies.

### Animal studies

All the animal studies were performed in accordance with the ARRIVE guidelines and the 3 Rs rule of Replacement, Reduction and Refinement principles. Mice were maintained and treated following the protocols approved by the CEEA (Ethical Committee for the Use of Experimental Animals) at the Vall d’Hebron Institute of Oncology, Barcelona, Spain. To generate tumors in the NSCLC model, 8- to 10-week old *LSL-Kras*^*G12D*^*;p53*^*ER/ER*^ mice were anesthetized with isoflurane (5%) and 30μL of EMEM + 12mM CaCl_2_ containing 5x10^7^ pfu of Ad-*Cre* were administered intranasally. Tumors in the PDAC model (*LSL-Kras*^*G12D*^*;Pdx1-Cre;p53*^*ER/ER*^) were spontaneously generated by tissue-specific expression of CRE recombinase. Mice of both models had a C56BL6/FVBN mixed background. 8-week old and 10 weeks post-AdCRE infection, for PDAC and NSCLC models respectively, mice initiated the 4-week treatment. The animals were treated twice a week (first and fourth day) by oral gavage with BAY 1238097 (35mg/kg) or an equivalent volume of NaCl 0.9% (vehicle) for 4 weeks. Weights were measured before every treatment and, in case of 10% cumulative weight loss relative to the treatment onset, the regimen was interrupted until weight was recovered. After the 4-week treatment, the animals were euthanized with CO_2_. Either lungs or pancreata were collected, fixed in 4% paraformaldehyde (PFA) and paraffin-embedded for histological analysis.

### Histology and sample analysis

Tissue sections were H&E-stained to quantify tumor burden (see Supplementary Figure 1). Accurate quantifications of tumor and normal tissue areas in the PDAC model were performed using 4 representative microscopy images of each section. In the NSCLC model, tumor and normal epithelium areas were quantified using the whole section. All areas were quantified using ImageJ. Percentage of tumor tissue was obtained by dividing the tumor area by the total area (tumor area + normal tissue area). Tumor grades of both models were blindly rated by a pathologist. Grades were evaluated considering previously published characterizations of both models [23, 24]. Representative images of each tumor grade are provided as Supplementary Figure 5.

### Cell lines

H1299, H441 and A549 were purchased from ATCC. HOP-62 was purchased from NCI. H460 and H23 were kindly donated by Dr. Aniello Cerrato and all PDAC cells were a gift from Dr. Silvestre Vicent Cambra. Cell lines (with the exception of HPAF-II, NP18 and MIA PaCa-2) were maintained in RPMI supplemented with 10% of FBS and 1% of glutamine. HPAF-II and NP18 were maintained in DMEM supplemented with 10% FBS and 1% of glutamine and MIA PaCa-2 with additional 2.5% horse serum. Murine cell lines were derived from the genetic mouse models of NSCLC (*LSL-Kras*^*G12D*^*;p53*^*ER/ER*^) and PDAC (*LSL-Kras*^*G12D*^*;Pdx1-Cre;p53*^*ER/ER*^) when adenocarcinomas were fully developed and the presence of the KRAS(G12D) mutation was confirmed (Supplementary Figure 3). Murine cells were maintained in DMEM/F12 containing 10% of FBS and 1% of glutamine.

### *In vitro* efficacy studies

PDAC- and NSCLC-derived cell lines from the *LSL-Kras*^*G12D*^*;Pdx1-Cre;p53*^*ER/ER*^ and *LSL-Kras*^*G12D*^*;p53*^*ER/ER*^ genetic mouse were seeded in 96-well plates at 500 cells/well. After 24 hours, cells were treated with increasing concentrations of BAY 1238097: 0.008, 0.016, 0.031, 0.063, 0.125, 0.250, 0.5, 1, 2 and 4 μM. 3 days after treatment, cells were fixed with PFA 4% for 20 minutes and stained with 0.5% crystal violet. Wells were washed twice using tap water and let dry for 24 hours. Staining was dissolved in 10% acetic acid and absorbance measured at 560nm to determine cell density. IC50s were determined using the XY Dose-response stimulation equation/log(dose) vs. response option of Graphpad Prism 7. Human cancer cell lines were seeded in 96-well plate at 1.000 cells/well. Cell density (relative to untreated controls) after 3 days of treatment at 0.63, 1.25, 2.5, 5, 10 and 20 μM was calculated using crystal violet staining and quantification of absorbance as previously described.

### Western blot

For mouse cells, 5×10^5^ cells were plated in 15cm dishes and treated after 24 hours with BAY 1238097 at 0.13 and 1.3 μM. 24 hours later, cells were scraped and harvested using cold PBS. Cells were lysed by resuspending the pellets in RIPA buffer containing Halt™ Protease Inhibitor Cocktail (Thermo) followed by a 30-minute incubation at 4°C and non-solubilized material was pelleted by centrifugation and discarded. Supernatants containing protein extracts were quantified by DC™ Protein Assay (Biorad) and equivalent protein concentrations dissolved in Laemmli buffer (containing 15% of β-mercaptoethanol). Samples were loaded in 15-well 10% Bis-Tris NuPAGE Novex Gels. The gel was run in MOPS buffer for 2 hours at 150V. Protein was transferred to a PVDF membrane using the standard procedure of the iBlot2 Dry Blotting System (Life Technologies). Membrane was stained using Ponceau to visualize total amount of protein as a loading control. Membrane was then blocked in 5% milk in PBS-0.1%Tween. MYC was detected using the anti-MYC Y69 antibody (Abcam) and tubulin using the anti-tubulin DM1A antibody (Sigma). Anti-rabbit and anti-mouse IgG-HRP (GE Healthcare) were used as secondary antibodies at 1:5,000. Membrane was incubated with Supersignal West Pico Chemiluminescent Substrate (Thermo) for 5 minutes before revealing. For human cell lines, each one was seeded at 1×10^6^ cells in 15cm dishes and, after 24 hours, either treated with 0.63 μM of BAY1238097 or left untreated. After 24 hours of treatment, cells were harvested as previously described. The following procedure is equivalent to the one applied to the mouse cells. ImageJ quantification of the MYC bands normalized by total protein content (Ponceau staining) was calculated to quantify MYC downregulation (see Supplementary Figure 4).

### Statistical analysis

All data were analyzed using GraphPad Prism 7.0 software. Statistical analysis was performed by two-tailed unpaired Student’s *t*-test and results were considered significant when p<0.05. Results are shown as mean ±SD. When mean was calculated using means of different cell lines (see Figure 4), mean ±SEM was represented.

## SUPPLEMENTARY MATERIALS FIGURES AND TABLE


