# Supplementary material for: BET inhibition is an effective approach against KRAS-driven PDAC and NSCLC
Source: Oncotarget. 2018 Apr 10;9(27):18734–46. doi: 10.18632/oncotarget.24648 (PMC5922351; doi:10.18632/oncotarget.24648)
Supplement: Supplementary file 1 [file oncotarget-09-18734-s001.pdf]

BET inhibition is an effective approach against KRAS-driven PDAC and NSCLC

SUPPLEMENTARY MATERIALS

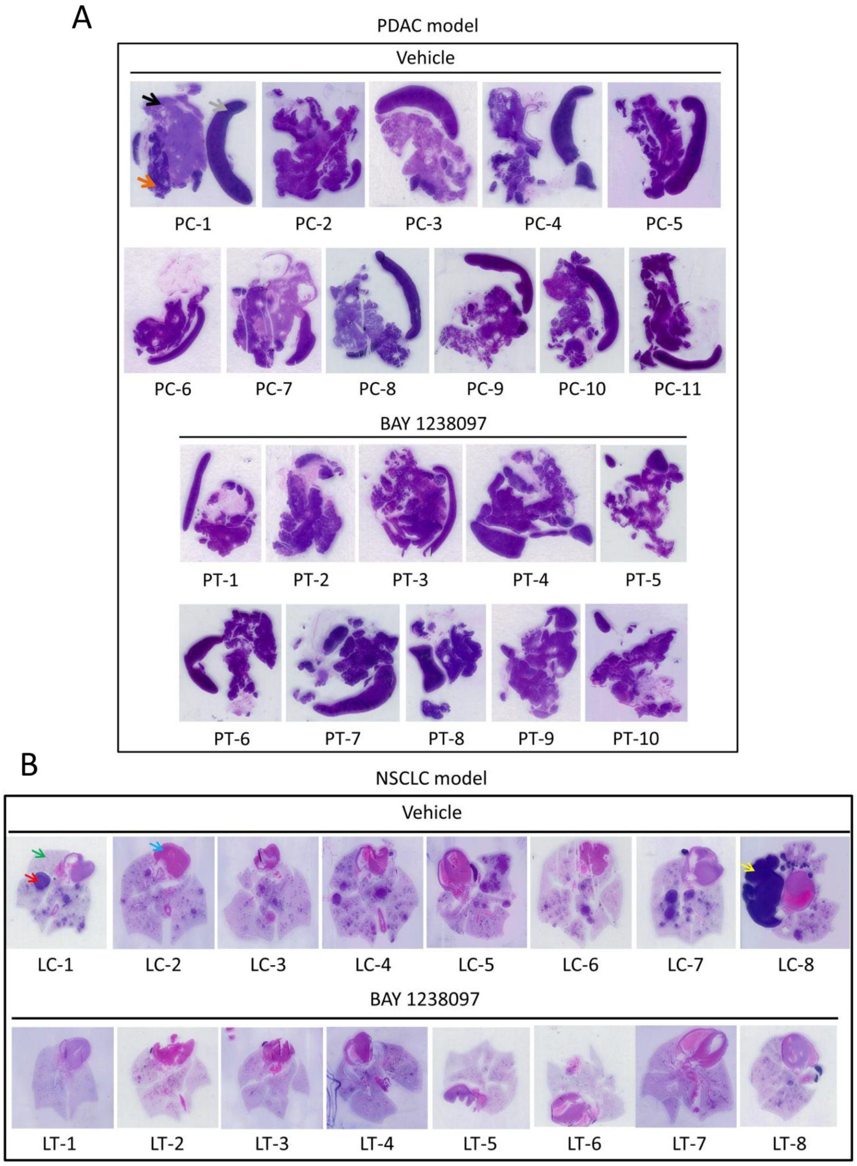

Supplementary Figure 1: BAY 1238097-treated animals (indicated with the letter "T") of the (A) PDAC and (B) NSCLC model present a reduction in tumor number and size compared to the vehicle-treated controls (indicated with the letter "C"). Tissue sections were collected and stained with H&E to visualize tumors and healthy tissue. None of the mice from the PDAC model were subjected to drug holiday due to weight loss. In contrast, LT-6 and LT-7 skipped one dose, and LT-8 two doses due to weight loss. LT-1, LT-3 and LT-6 show total absence of tumors. Arrows indicate examples of: pancreatic tumor (black); normal exocrine tissue (orange); spleen (grey); healthy lung epithelium (green); lung tumor (red); heart (blue) and thymoma (yellow). PC: pancreas control; PT: pancreas treated; LC: lung control; LT: lung treated

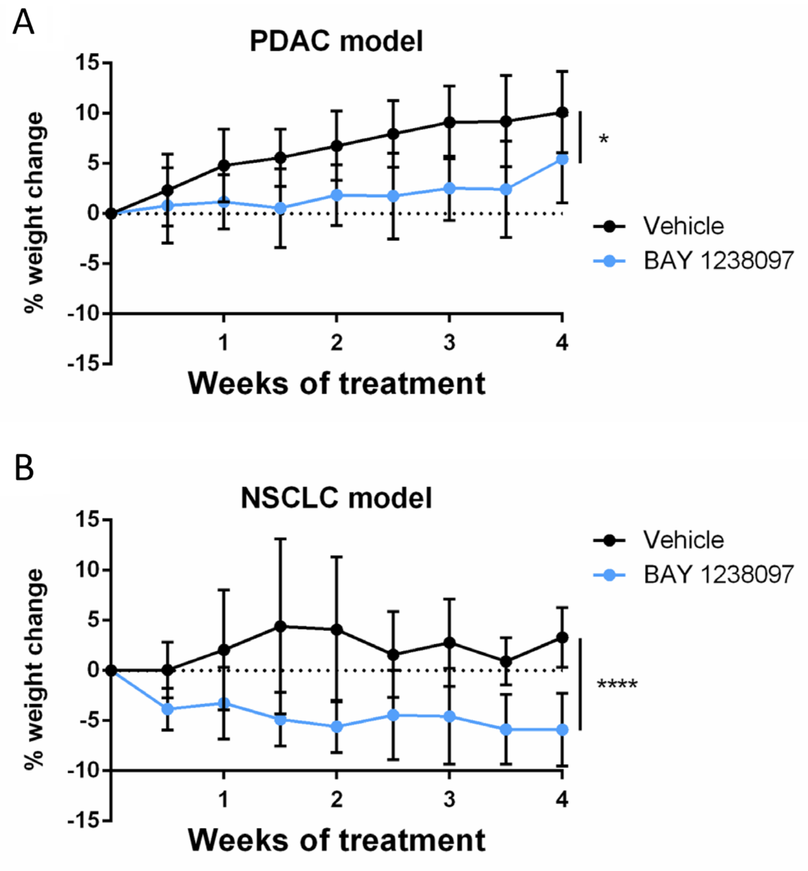

Supplementary Figure 2: BAY 1238097 causes a moderate but significant reduction in weight. **(A)** Weights of 8 to 12 week-old mice harboring PDAC and **(B)** 20 to 24 week-old mice harboring NSCLC, treated with the BET inhibitor or untreated. Graphs show changes in weight relative to the treatment onset. X axis have been placed at "-10%", the weight value representing the threshold of treatment interruption. Means and standard deviations are represented. Two-tailed unpaired *t*-tests were performed at treatment endpoint (4 weeks) to assess statistical significance: p=0.0176 (*) and p<0.0001 (****).

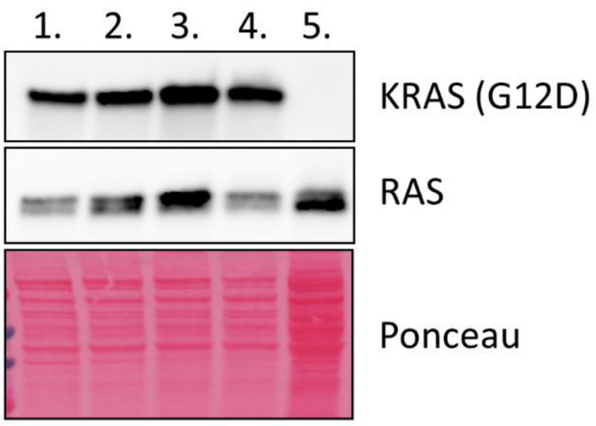

Supplementary Figure 3: The 4 murine cell lines derived from the genetic mouse models of PDAC and NSCLC harbor the tumor-initiating KRAS(G12D) point mutation. Clones derived from the NSCLC model (MLT#1 and MLT#6, lanes 1 and 2 respectively) and from the PDAC model (mPDAC1.1 and mPDAC1.2, lanes 3 and 4 respectively) were compared to primary mouse embryonic fibroblasts (lane 5) to confirm the expression of KRAS(G12D) in the established cancer cell lines. Anti-RAS (clone RAS10, Merck) to detect the expression of RAS proteins and a specific anti-RAS(G12D) (D8H7, Cell Signaling) to detect the point mutant were used. Ponceau staining is shown as protein loading control.

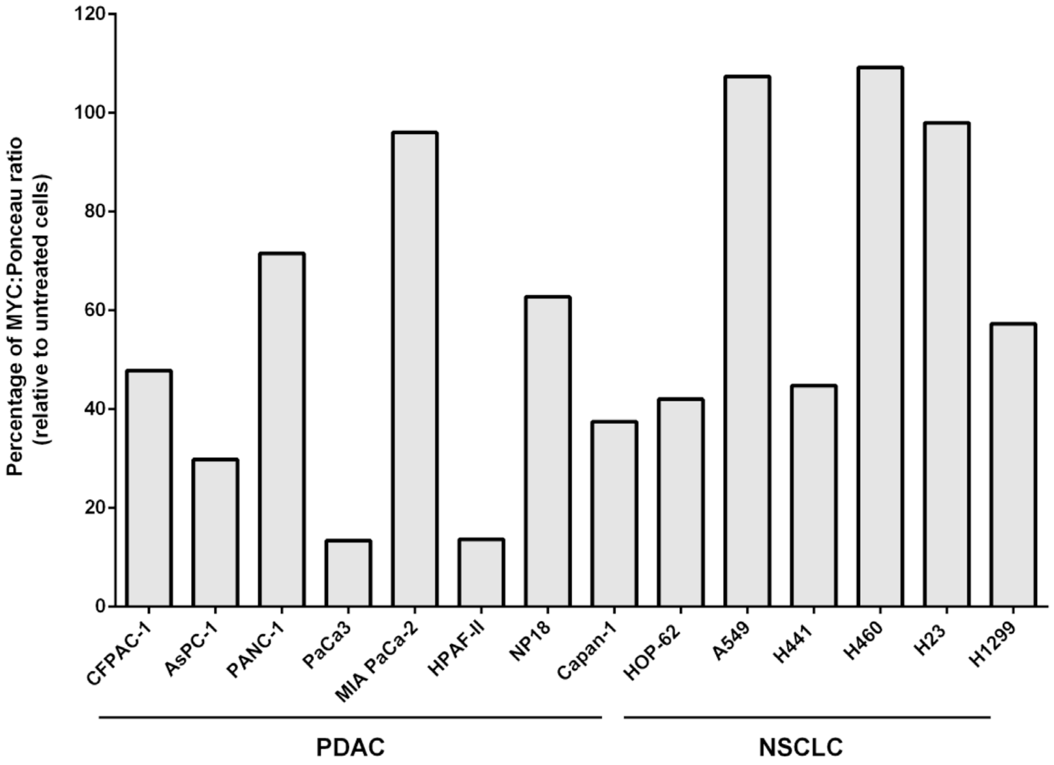

Supplementary Figure 4: Among the PDAC and NSCLC cell lines, CFPAC-1, AsPC-1, PaCa3, HPAF-II, Capan-1, HOP-62, and H441 show the most notable MYC downregulation (reduction to 50% or less). Graph shows percentage of treated: control band intensity. Quantification of Ponceau and MYC from Figure 3 has been performed using ImageJ. MYC levels were normalized by the Ponceau staining (total protein).

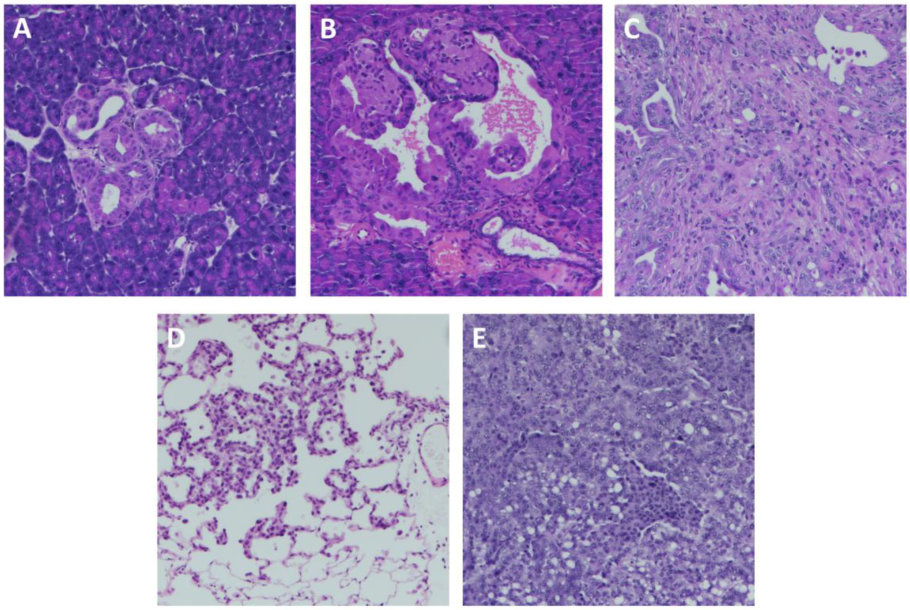

Supplementary Figure 5: Representative microscopic images of the different tumor grades found in the sections from PDAC and NSCLC models (Table 1). For PDAC, PanIN2 (A), PanIN3 (B) and PDAC (C) were found. For NSCLC, AAHs (D) and adenocarcinomas (E) were found; no large adenoma was found in the NSCLC sections. These grades were evaluated by a pathologist.

Supplementary Table 1: LKB1 and TP53 protein status of (A) NSCLC and (B) PDAC cell lines according to the COSMIC and ATCC databases

A		
**NSCLC – AA Mutation**		
**Cell line**	**LKB1**	**TP53**
HOP-62	wt	p.?
A549	p.Q37*	wt
H441	wt	p.R158L
H460	p.Q37*	wt
H23	p.W332*	p.M246I
H1299	wt	p.?

B		
**PDAC – AA Mutation**		
**Cell line**	**LKB1**	**TP53**
Capan-1	wt	p.A159V
CFPAC-1	wt	p.C242R
AsPC-1	wt	p.C135fs*35
PANC-1	wt	p.R273H
PaCa3	-	wt
MIA PaCa-2	wt	p.R248W
HPAF-II	wt	p.P151S
NP18	-	-

AA Mutation: aminoacidic mutation. wt: wildtype "*": nonsense substitution. "-": no information available. "p.?": unknown. "Fs": frameshift.
